# Impact of cavitron ultrasonic surgical aspirator (CUSA) and bipolar radiofrequency device (Habib-4X) based hepatectomy for hepatocellular carcinoma on tumour recurrence and disease-free survival

**DOI:** 10.18632/oncotarget.21271

**Published:** 2017-09-26

**Authors:** Kai-Wen Huang, Po-Huang Lee, Tomokazu Kusano, Isabella Reccia, Kumar Jayant, Nagy Habib

**Affiliations:** ^1^ Department of Surgery & Hepatitis Research Center, National Taiwan University Hospital, Taipei, Taiwan; ^2^ Centre of Mini-invasive Interventional Oncology, National Taiwan University Hospital, Taipei, Taiwan; ^3^ Graduate Institute of Clinical Medicine, College of Medicine, National Taiwan University, Taipei, Taiwan; ^4^ Department of Surgery and Cancer, Imperial College London, London, UK

**Keywords:** hepatocellular cancer, liver resection

## Abstract

**Background:**

The aim of this study was to evaluate the oncological outcomes of hepatocellular carcinoma patients undergoing liver resection using cavitron ultrasonic surgical aspirator (CUSA) or radiofrequency (RF) based device Habib-4X.

**Study Design:**

We prospectively analyzed the data of 280 patients who underwent liver resection for hepatocellular carcinoma at our institution from 2010–2012 with follow up till August 2016. The CUSA was used in the 163 patients whilst Habib-4X in 117 patients. The end points of analysis were oncological outcomes as disease recurrence, disease-free survival (DFS) and overall survival (OS) were estimated by the Kaplan–Meier method, which has been compared with all other existing literature on the survival study.

**Results:**

Compared with CUSA the reported incidence of recurrence was significantly lower, in Habib-4X group; *p* < 0.01. The median DFS was significantly better in Habib-4X group than CUSA group (50.80 vs 45.87 months, *p* = 0.03). The median OS was better in Habib-4X group than CUSA group (60.57 vs 57.17 months, *p* = 0.12) though the lesser difference in OS between the groups might be explained by the use of palliative therapies as TACE, percutaneous RFA, etc. in case of recurrence.

**Conclusions:**

RF based device Habib-4X, is safe and effective device for resection of hepatocellular carcinoma, in comparison to CUSA with better oncological outcomes, i.e., significantly lesser tumour recurrence and better DFS. This could be explained on the basis of systemic and local immunomodulatory effect involving induction of kupffer cells and effector CD-8 T cells that help in minimizing postoperative complications and bring more advantageous oncological outcomes.

## INTRODUCTION

Hepatocellular carcinoma (HCC) has been reported as the fifth most common malignancy and considered as one of the aggressive cancer of mankind. In 2012, the worldwide incidence of HCC was 782,000 with mortality of 746,000 per year [1]. In the present era, surgical resection of the tumour has been considered as the standard treatment option for early Barcelona classified HCC patients. Moreover, the indication for surgical resection has widened owing to improved knowledge of liver anatomy, anaesthetic techniques, intraoperative ultrasounds and other imaging techniques [2, 3]. In the last two decades surgical world has witnessed the introduction of many devices to ease the technical challenges imposed with liver resection, though the device of choice has remained an issue of debate till date.

The initial prototype technique of liver resection is clamp crush or finger fracture is associated with high incidence of intraoperative bleeding during parenchymal transection and is the major obstacle to surgical success [4]. Excessive blood loss and blood transfusions have further ratified the perioperative morbidity, infections and mortality. Furthermore, it is associated with an increased risk of HCC recurrence [5, 6]. To limit the blood flow during parenchymal transection an infamous hepatic vascular inflow occlusion technique or Pringle manoeuvre has been introduced into the practice although its applicability is limited particularly in patients with underlying liver disease owing to an increase risk of ischemic reperfusion injury and inability to control back-flow bleed from hepatic veins [7, 8].

Early era of liver surgery has evidenced a mortality of 10-20%, which significantly reduced to 5% with further advancements in the medical science, equipment and operative techniques [9]. Various equipments’ of liver resection, such as the CUSA (Cavitron Ultrasonic Surgical Aspirator), RF based Habib-4X, Ligasure (Valley Lab, Tyco Healthcare, Boulder, CO, USA), Harmonic Scalpel (Ethicon Endo-Surgery, Cincinnati, OH, USA), TissueLink (Salient Surgical Technologies, Portsmouth, NH, USA), Water-jet dissection, microwave assisted resection, vascular staplers, and others have been introduced to facilitate easy and safe resection of liver parenchyma. Despite that, the question regarding the clinical benefit of using one over the other still remains unanswered [10].

Cavitron Ultrasonic Surgical Aspirator (CUSA), also known as Ultrasonic Dissector has been first popularized by Hodgson et al. in 1979 [11]. Here, the ultrasonic waves generate energy to fragment and aspirate parenchymal tissue. The contact of oscillating titanium tip causes fragmentation of hepatocytes owing to the high water content while, selectively sparing the blood vessels and bile ducts because of poor tissue water content. As CUSA doesn’t coagulate, one needs additional help of ties, clips or staplers as per the surgeon’s disposition to achieve haemostasis and biliostasis. Consequently, it results in an explicit line of transection by safeguarding the normal hepatic tissue, though the benefit obtained in terms of reducing the blood loss is not significant [12–14]. The ability of CUSA to selectively modulate tissue dissection depends upon the mechanical resistance offered by the tissue itself, e.g., hepatocytes contain less fibrous tissue than vessels and thus extend less resistance to crushing during parenchymal division. This is a particularly important point for consideration in a cirrhotic liver by virtue of an increased fibrous component [14–16].

Habib’s technique, first introduced by Habib in 2002, has received well acceptance as “Bloodless Hepatectomy Technique”. The Habib-4X is a bipolar device introduced perpendicularly into the liver in a serial fashion to create a parallel lines of ablation [17, 18]. This RF based device permits hepatic resection with minimal blood loss [19]. It coagulates all vessels and bile ducts in its field of application thus minimizing the need of Pringle Maneuver and blood transfusion [20].

The present study is the first and largest study done so far to compare the oncological outcomes of CUSA and Habib-4X based liver resection. Based on the prospective analysis of our database, we specifically compared the recurrence rates, DFS and OS following use of these two modalities.

## RESULTS

### Demography

A total of 280 patients with hepatocellular carcinoma who underwent hepatic resection were included in the present study. CUSA based hepatic resection was performed in 163 patients while 117 patients were treated with Habib-4X. Patients’ demographic characteristics of each group has been tabulated (Table 1) and compared. The mean age of patients in CUSA and Habib-4X group was 58.39 ± 11.9 years and 58.18 ± 11.37 years respectively (*p* > 0.05). There were 31 women (19.0%) and 132 men (80.9%) in the CUSA cohort whilst, 13 women (26.4%) and 86 (73.5%) men in the Habib-4X group. Along with that, we didn’t observe any significant differences between groups regarding serum albumin, serum bilirubin, serum AFP, tumour numbers, tumour size, tumour stage, cirrhosis, HBsAg, HCV, ICG clearances (Tables 2 and 3).

**Table 1:** Demographics and clinical characteristics of patients in the study groups

Parameters	CUSA (163)	Habib-4X (117)	*p* value
Mean age ±SD (yrs)	58.39 ± 11.9	58.14 ± 11.37	0.85
No. male/female	132/31	86/31	0.14
Albumin (g/dl)	4.28 ± 0.88	4.05 ± 1.20	0.08
Bilirubin (mg/dl)	1.06 ± 0.86	.97 ± 0.49	0.27
ICG Clearance (15 mins)	9.41 ± 7.2	10.62 ± 9.4	0.22
AFP (ng/ml)	1790.02 ± 7623.98	1901.17 ± 9763.70	0.91
Cirrhosis	64	51	0.47
HBsAg	112	78	0.72
HCV	46	40	0.29

• Statistical significance was analyzed by the chi-square test.

• Statistical significance was analyzed by the Student’s *t*-test

**Table 2:** Tumour characteristics of patients in study groups

Parameters	CUSA (163)	Habib-4X (117)	*p* value
Tumour Numbers			
1	144	106	0.68
2	13	9	0.93
3	4	1	0.58
4	2	1	0.76
Tumour Stage			
T1	106	79	0.76
T2	53	34	0.63
T3	4	4	0.91
Tumour Size (cm)	4.66 ± 3.43	4.43 ± 3.27	0.56

• Statistical significance was analyzed by the chi-square test.

• Statistical significance was analyzed by the Student’s *t*-test.

**Table 3:** Operative and postoperative characteristics of patients in study groups

Parameters	CUSA (163)	Habib-4X (117)	*p*-value
Anatomical resection	63	43	0.84
Non-anatomical resection	100	74	0.84
Major resection	45	27	0.43
Minor resection	116	90	0.43
Blood Loss (mL) (Mean ± SD)	271.47 ± 214.5	150.93 ± 103.6	0.00*
Blood transfusion received	21	3	0.00^#^
Vascular Inflow Control	139	1	0.00^#^
Hospital Stay (Days) (Mean ± SD)	7.84 ± 2.04	7.88 ± 1.25	0.77
Post hepatectomy liver failure	4	0	
Bile leakage	4	1	
Sepsis	0	2	
Bleeding	1	0	
Major complication	9	3	0.36

• *^#$^ Showing Significant *p*- value.

• ^#^Statistical significance was analyzed by the chi-square test.

• *Statistical significance was analyzed by the Student’s *t*-test.

• ^$^Statistical significance was analyzed by the Mann-Whitney *U* test.

### Procedure and complications

No significant difference was observed in the number and size of tumours in the CUSA and the Habib-4X resection groups (Table 2). Major liver resection was done for 45 patients in CUSA group and 27 patients in Habib-4X group with no significant differences (*p* = 0.43). Anatomical resection was accomplished in 63 cases with CUSA while 43 cases were done with Habib-4X (*p* = 0.84) (Table 3)

The operative blood loss was significantly higher (271.47 ± 214.5 mL) in the CUSA group than Habib-4X group (150.93 ± 103.6 mL; *p* < 0.00). Furthermore, our results demonstrated single event of vascular inflow control in Habib-4X group compared to 139 in the CUSA group (*p* < 0.00; Table 3).

The length of postoperative hospital stay was comparable for patients in both the groups. The mean length of stay was 7.88 ± 1.25 days (5–12 days) in the Habib-4X group compared to 7.84 ± 2.04 days (6–21days) in CUSA group (*p* = 0.77). We reported 0% mortality as in-patient or within 30 days of hospital admission in both groups (Table 3).

The major complications as post hepatectomy liver failure, bile leakage, bleeding were higher in CUSA group than Habib-4X although not reached any statistical significance (*p* = 0.36; Table 3).

The data analysis of tumour histology showed that free resection margins were comparable in both groups i.e., 91 cases with CUSA than 57 cases in Habib-4X group (*p* = 0.29) also vascular invasion were comparable (Table 5).

### Recurrence

We registered significantly lower recurrence in the Habib-4X group (44 patients) as compared to the patients treated with CUSA (85 patients; *p* < 0.01) (Table 4). Similarly, the percent of patients who received other palliative interventions post recurrences were significantly higher for the CUSA group i.e., 81.1% compared to 62.4% in Habib-4X group (*p* < 0.01) The further analysis of the data showed RF ablation as the most common modality of palliation in CUSA group (47/85; 55.3%) compared to Habib-4X (8/44; 18.1%); The TACE was the second most common modality of palliation required in 23/85; 27% cases of CUSA group compared to 21/44; 47.7% cases in Habib-4X group (Table 4).

**Table 4:** Comparison with number of interventions post-recurrence within studied groups

Parameters	CUSA (163)	Habib-4X (117)	*p* value
Recurrence (−/+)	78/**85**	73/**44**	0.01^#^
Intervention done in cases of recurrence (−/+) (%)	78/**85** **(81.1%)**	73/**44** **(62.4%)**	0.00^#*^
RFA	47	8	
TACE	23	21	
Re-operation	9	3	
Sorafenib	5	12	
Radiotherapy	1	0	

• ^#^*Showing Significant *p*-value.

• *Degree of freedom = 5.

• ^#^Statistical significance was analyzed by the chi-square test.

**Table 5:** Postoperative tumour characteristics of patients in study groups

Parameters	CUSA (163)	Habib-4X (117)	*p*-value
Resection margin			
Free	91	57	0.29
Free within 1 cm	63	52	0.39
Involved	9	6	0.82
Vascular invasion	42	27	0.67
Local Recurrence	5	3	0.80^#^
Recurrence (−/+)	78/85	73/44	0.01^#^
Disease Free Survival (Median) (months)	45.87	50.80	0.03^$^
Overall Survival (Median) (months)	57.17	60.57	0.11

• ^*#$^Showing Significant *p*- value.

• ^#^Statistical significance was analyzed by the chi-square test.

• *Statistical significance was analyzed by the Student’s *t*-test.

• ^$^Statistical significance was analyzed by the Mann-Whitney *U* test.

### Survival

**Figure 1:**
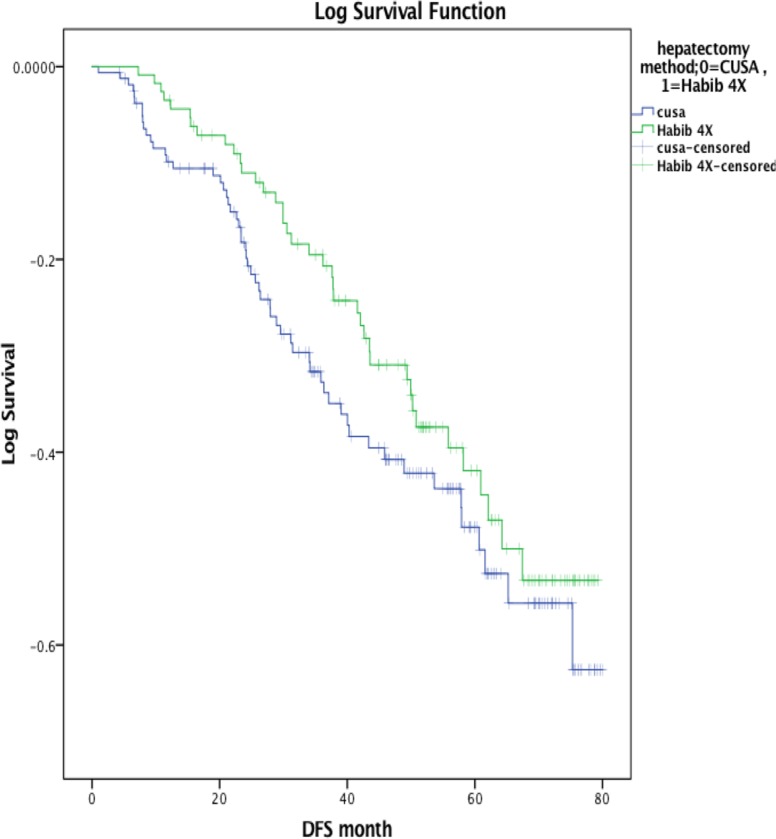
Disease-free survival (DFS) estimates Kaplan-Meier survival plot of DFS comparing CUSA group (Blue line) with the Habib-4X group (Green line) (*p* = 0.03).

The median duration of disease-free survival was significantly longer in the Habib-4X group (50.80 months) than CUSA group (45.87 months; *p* = 0.03). Five-year disease free survival was 56% in the Habib-4X group compared to 54% in the CUSA group while the corresponding overall survival rates for the Habib-4X group and CUSA group were 61% and 51.30% respectively (Table 5; Figure 1). Although, the median overall survival was better in Habib-4X group than CUSA group but was not statistically significant (60.57 vs 57.17 months, *p* = 0.12) (Table 5).

## DISCUSSION

Studies have proved that surgical resection confers better likelihood of cure and survival in patients suffering from early stage of HCC [21, 22]. Nevertheless, disagreement exists regarding the most efficacious and safest modality of liver resection. Current evidence indicates that the stage of parenchymal transection during surgery has maximum impact on blood loss, blood transfusion, post-operative complications and bile leak. Furthermore, this has also influenced the oncological outcomes such as survival and tumour recurrence [23–26]. Studies have shown that Habib-4X provides favourable operative outcomes in terms of blood loss, post-operative recovery, hospital stay, postoperative morbidity and mortality [17, 27, 28]. Till date, many studies have looked into the safety and efficacy of various modalities for hepatic parenchymal resection, however very few studies have compared the long-term outcomes of these techniques [29]. The disease recurrence and survival are considered as the most important factors determining the therapeutic success in management of malignant disease. To the best of our knowledge, this is the first study to investigate into the oncological outcomes following the use of CUSA or Habib-4X for liver resection. Here we matched and compared both groups to minimize confounders as serum albumin, serum bilirubin, serum AFP, tumour numbers, tumour size, tumour stage, cirrhosis, HBsAg, HCV, ICG clearances, type of resection (major/minor and anatomical/non-anatomical), resection margin, tumour number, size and stage, and vascular invasion. The data analysis showed that both groups were comparable against above outlined parameters with no significant difference (*p* < 0.05) and has increased significant strength to our study.

The analysis of immediate outcomes in our study showed that the mean blood loss, need for blood transfusion, and requirement for vascular inflow control was significantly lower in the Habib-4X group than the CUSA group. This might be explained by the fact that Habib-4X utilizes radiofrequency energy to coagulate the blood vessels while the ability of CUSA has been limited by the need of manual activation to coagulate the blood vessels [30–33]. In accordance with a previous study, we also found that surgeon’s experience in using CUSA has an influence on the surgical outcome [34].

The length of hospital stay did not reach statistical significance in either study group, however this appeared similar to other existing reports. The present study didn't report any mortality in either group which could be attributed to the recent advances in the surgical techniques and better peri-operative and post-operative management making hepatic resection a reasonably safe treatment option.

As, recurrence following curative surgery in patients with HCC remains a major clinical hurdle while deciding the best treatment strategy. We demonstrated significantly lower recurrence with the RF based device Habib-4X. One of the reasons accounting for the present oncological outcomes are based on the coagulating property of RF ablation which minimises blood loss and the need for blood transfusion; prevents any tumour spillage and micro metastasis. The lower recurrence could also be attributed to the favourable systemic and local immunomodulatory changes induced by the RF resection [34–36].

The further data analysis of long term outcomes has demonstrated significantly better disease-free survival for the patients who underwent RF based liver resection for HCC. The disease-free survival rates were 95% at 1 year, 96% at 2 years, 88% at 3 years, 67% at 4 years and 56% at 5 years, (Table 6) which was not only better than the CUSA group but also stands in accordance with the previous notable studies [23, 37–53]. We also evidenced better overall survival in the Habib-4X group, although, it did not reach statistical significance. This might be because the recurrence of cancer did affect the disease free survival though this was not reflected in terms of overall survival. The inability to reach statistical significance could be explained by the higher use of palliative treatment modalities like percutaneous RF ablation, TACE, sorafenib etc. in the CUSA group in instances of recurrence which helps in prolonging life despite recurrence of the disease. This has facilitated the importance of Habib-4X in providing better disease free survival and this further supports the recently published study Qiu et al. where they reported significantly better recurrence free survival and overall survival with Habib-4X compared to the clamp-crush technique [54].

**Table 6:** Detailed comparison of disease free survival for the study groups

Parameters (Time in Months)	CUSA (163)	Habib-4X (117)
12 months	93%	95%
24 months	87%	96%
36 months	86%	88%
48 months	66%	67%
60 months	54%	56%

Following initial hepatic resection, 81.1% patients in CUSA group while 62.4% in Habib-4X required retreatment. Further analysis of data showed that RF ablation was the most common modality of palliation (55.3%) in the CUSA recurrence group while it constituted only 18.1% of palliation in the Habib-4X group. The findings further strengthened the role of Habib-4X as a better modality of treatment over CUSA in HCC management.

The favourable oncological outcomes with Habib-4X could be explained by the virtue of systemic and local immunomodulatory effect of radiofrequency. The debris produced following RF-induced coagulative necrosis during HCC resection releases tumour antigens and chemokines. These chemokines attract inflammatory infiltrates; neutrophils, macrophages, NK cells, dendritic cells (DCs), as well as CD4+ and CD8+ T lymphocytes. The cellular influx at the ablated resection margin phagocytoses the debris and tumour cells. Tumour antigens also drain to nearby lymph nodes, and stimulate immature DCs and naive T-cells thus provide systemic immunomodulation [55–58].

The liver is a unique organ which maintains intricate balance between not over-reacting to the antigens absorbed by the gut and mounting accurate immune responses to eliminate the tumor antigen. HCC is characterized by chronic inflammation and immune suppression. Immunosuppression due to inhibitory checkpoints appear to be an important contributor to the induced immune suppression in this setting and subsequent development and progression of HCC. Tregs and myeloid-derived suppressor cells are thought to play an important role in protecting the tumour from eradication by activated cytotoxic CD8+ T cells. High level of Tregs in tumor has been linked to poor prognosis in HCC [59–62].

Over the last two years, major breakthrough in immunological understanding in the tumour management has led to the development of newer drugs as checkpoint inhibitors which boost CD8+ T cell functioning. The discovery of these drugs have brought significant improvement in the survival of patients with cancers like leukaemia, lymphoma and melanoma, although the same has not been translated for HCC. One of the great potentials of RF based therapies are that they can dovetail with immune modulating therapies. RF ablation induces CD8+ T cells infiltration at the ablation site and further addition of a checkpoint inhibitor might act as a booster. The potential effect on the immune system is further advantageous in terms of better survival as it acts in synergy with checkpoint inhibitors. In addition, recent trials have shown that combined use of RF and checkpoint inhibitors could bring more beneficence towards the long-term survival [63–66]. This has been further strengthened by the study of Duffy et al., 2017, where they reported the activation of the immune system through checkpoint inhibitors and accumulation of intratumoral CD8+ T cells following RF ablation, thus demonstrating the synergism of combined use of checkpoint inhibitor tremelimumab and ablation in aggressive hepatocellular carcinomas [67].

The present study has certain limitations owing to its retrospective design and unintended biases of patient selection which might influence the analysis. In spite of these limitations, this study has outlined significantly better disease free survival and lesser tumour recurrence with the Habib-4X group compared to the CUSA group.

## MATERIALS AND METHODS

### Study design

In this multicentric study, the data from two centers of National Taiwan University Hospital were prospectively collected and analyzed after obtaining approval from the Institutional Review Board. The data included 280 patients with confirmed diagnosis of HCC on histopathology who underwent liver resection with CUSA or RFA based device Habib-4X from January 2010 till December 2012 and were followed-up till August 2016. The data was collected for the amount of blood loss, vascular inflow control, length of hospitalization, complication within 30 days, post hepatectomy liver failure (PHLF) overall recurrences, local recurrences and interventions done in cases of recurrences. PHLF was measured according to the definition set by International Study Group of Liver Surgery (ISGLS) 2011 [68]. The endpoints of the study as overall survival and disease free survival were estimated by the Kaplan–Meier method.

### Procedure

All patients with a diagnosis of HCC underwent open surgical hepatectomy under guidance of intra-operative ultrasound. Both the lobes of liver were mobilized and gall bladder was removed if needed. Selective inflow control was performed in cases of excessive parenchymal bleeding. In cases where CUSA had been used by the surgeon, help of an assistant surgeon was needed to control the bleeding with bipolar coagulation, while no assisting haemostatic instrument was required during resection in the Habib-4X group. The Habib-4X, a bipolar device was introduced perpendicularly into the liver in a serial fashion to create parallel lines of ablation. The third line of ablation was created perpendicular to the parallel track, following which liver was resected by scalpel. The probe was moved swiftly in see-saw fashion for 3-5 mm in its axis of application. The movement of probe helped in averting any adherence of the liver tissue. The device effectively created a 1 cm thick area of ablated and coagulated tumour free margin [16, 17]. A meticulous hemostasis was assured, and raw surface covered by cellulose hemostatic agent. In the present study, a major hepatectomy was defined as resection of three or more liver segments.

### Statistical analysis

Overall survival and disease-free survival were calculated from the date of surgical intervention. Continuous variables were analyzed with Student’s *t*-test, and categorical variables were analyzed with chi-square or Fisher’s Exact Test where appropriate. Survival and recurrence rates were calculated using the Kaplan- Meier method and comparison between groups were done with the log-rank test. Predictors of overall and disease-free survival were analyzed by performing a Cox Proportional Hazards regression model using a backwards selection process. A *p-* value of < 0.05 was considered significant in this study. Data were fed into a Microsoft Excel worksheet and analyzed by the IBM-SPSS version 24 (SPSS Inc, Chicago, IL, USA).

### Literature review

A comprehensive systematic literature review was performed to search all the published articles on National Library of Medicine Database (PUBMED), EMBASE, Cochrane, CrossRef, and Scopus databases on 15th November 2016 describing the outcomes of hepatic resection in hepatocellular carcinoma. The search covered the period from January 1st, 1981 to November 15th, 2016. The search was carried out by using the medical subject headings (MeSH) terms: ‘Hepatocellular Carcinoma’, ‘Liver Neoplasm’, ‘Hepatectomy open’, ‘Hepatectomy laparoscopic’. The initial search yielded a total of 154 manuscripts. Following the careful evaluation of inclusion, exclusion criterias and demography characteristics, 137 articles were excluded. The remaining 17 papers, were considered, and full-text obtained (Table 7).

**Table 7:** Literature review of survival outcomes of hepatic resection in hepatocellular carcinoma

Author	Year	Study Sample	Hospital Mortality (%)	Median Survival (month)	Overall Survival (%)		
					1 year	3 year	5 year
Heng-Jun et al. [37]	2014	151	-	61.8	99	68	52
Jin et al. [40]	2014	62	11.1	-	83.2	75.7	65
Jianyong et al. [38]	2014	433	2.3	-	91.8	84.2	70.8
Nojiri et al. [41]	2014	107	-	-	-	62	38.1
Lim et al. [42]	2014	172	1	-	-	-	58
Yin et al. [43]	2014	88	11.3	41	76.1	51.5	-
Zhong et al. [21]	2014	660	2.6	-	91	67	44
Zhong et al. [44]	2013	257	3.1	42.9 ± 26.1	84	59	37
Cheng et al. [45]	2012	104	7.3	-	90	-	66
Hsu et al. [46]	2012	268	2.7	-	81	63	43
Lin et al. [52]	2010	93	5.4	29.9±20.1	83	49	-
Ho et al. [47]	2009	294	-	37.9	77.4	51.9	36.6
Wang et al. [48]	2008	243	-	60.4±6.1	81.5	64.4	50.5
Lee et al. [49]	2007	100	2	-	66	44	31
Pandey et al. [53]	2007	166	3	-	-	-	29
Ng et al. [50]	2005	380	2.7	-	74	50	39
Poon et al. [51]	2002	120	5	-	61	38	28
Present Study	2016	117	1.5	60.6	95	86	56

## CONCLUSIONS

The present study reported better oncological outcomes including significantly lesser tumour recurrence and better disease free survival following Habib-4X based tumour resection for hepatocellular cancer as compared to CUSA.

The small difference in terms of OS between the two groups could be explained by the use of palliative therapeutic modalities in the patients with tumour recurrence. Habib-4X is a feasible, promising and safe liver resection device with excellent short and long term results and with a potential to be used with checkpoint inhibitors. Nevertheless, these findings need to be confirmed with more prospective and randomized controlled trials.
